# Sex ratio in dementia with Lewy bodies balanced between Alzheimer’s disease and Parkinson’s disease dementia: a cross-sectional study

**DOI:** 10.1186/s13195-018-0417-4

**Published:** 2018-09-12

**Authors:** A. Mouton, F. Blanc, A. Gros, V. Manera, R. Fabre, E. Sauleau, I. Gomez-Luporsi, K. Tifratene, L. Friedman, S. Thümmler, C. Pradier, P. H. Robert, R. David

**Affiliations:** 1 Université Côte d’Azur, CobTeK lab, Nice, France; 2 Centre Mémoire de Ressources et de Recherche, Institut Claude Pompidou, 10 rue Molière, 06100 Nice, France; 3 Geriatrics Department, University Hospitals of Strasbourg, CMRR (Research and Resources Memory Centre), Geriatric Day Hospital, Strasbourg, France; 4 University of Strasbourg and CNRS, ICube Laboratory UMR 7357 and FMTS (Fédération de Médecine Translationnelle de Strasbourg), Team IMIS/Neurocrypto, Strasbourg, France; 5 Centre Hospitalier Universitaire de Nice, Department of Public Health, L’Archet Hospital, Nice University Hospital, EA 6312 Nice, France; 6 University of Strasbourg and CNRS, ICube Laboratory UMR 7357, Strasbourg, France; 7 Bastia Hospital, Memory Center, Bastia, France; 8 Antibes Hospital, Memory Center, Antibes, France; 9 Veterans Affairs Palo Alto Health Care System, Palo Alto, CA USA; 10 Department of Psychiatry and Behavioral Sciences, Stanford School of Medicine, Stanford University, Stanford, CA USA; 11 Centre Hospitalier Universitaire de Nice, University Department of Child and Adolescent Psychiatry, Children’s Hospitals CHU-Lenval, Nice, France

**Keywords:** Dementia with Lewy bodies, Alzheimer’s disease, Parkinson’s disease, Parkinson’s disease dementia, Sex ratio

## Abstract

**Background:**

Gender distribution varies across neurodegenerative disorders, with, traditionally, a higher female frequency reported in Alzheimer’s disease (AD) and a higher male frequency in Parkinson’s disease (PD). Conflicting results on gender distribution are reported concerning dementia with Lewy bodies (DLB), usually considered as an intermediate disease between AD and PD. The aim of the present study was to investigate gender differences in DLB in French specialized memory settings using data from the French national database spanning from 2010 to 2015 and to compare sex ratio in DLB with that in AD, Parkinson’s disease dementia (PDD), and PD. Our hypothesis was that there is a balanced sex ratio in DLB, different from that found in AD and PD.

**Methods:**

We conducted a repeated cross-sectional study. The study population comprised individuals with a DLB, AD, PDD, or PD diagnosis according to the International Classification of Diseases, Tenth Revision, in the French National Alzheimer Database between 2010 and 2015. Sex ratio and demographic data were compared using multinomial logistic regression and a Bayesian statistical model.

**Results:**

From 2010 to 2015 in French specialized memory settings, sex ratios (female percent/male percent) were found as follows: 1.21 (54.7%/45.3%) for DLB (*n* = 10,309), 2.34 (70.1%/29.9%) for AD (*n* = 135,664), 0.76 (43.1%/56.9%) for PD (*n* = 8744), and 0.83 (45.4%/54.6%) for PDD (*n* = 3198). Significant differences were found between each group, but not between PDD and PD, which had a similar sex ratio.

**Conclusions:**

This large-sample prevalence study confirms the balanced gender distribution in the DLB population compared with AD and PD-PDD. Gender distribution and general demographic characteristics differed between DLB and PDD. This is consistent with the hypothesis that DLB is a distinct disease with characteristics intermediate between AD and PD, as well as with the hypothesis that DLB could have at least partially distinct neuropathological correlates.

## Background

Dementia with Lewy bodies (DLB) is the second leading cause of neurodegenerative dementia, representing 10–15% of dementia cases [16]. DLB is characterized by fluctuating cognitive deficits, visual hallucinations, REM sleep behavior disorder (RBD), and parkinsonism [17]. Clinical aspects are close to those of Alzheimer’s disease (AD), with impairments in episodic memory and/or executive dysfunction, but they are also close to those of Parkinson’s disease (PD), with the presence of motor alterations. The term *Parkinson’s disease dementia* (PDD) is used when dementia appears more than 1 year after the onset of typical PD, whereas the term *dementia with Lewy bodies* refers to a dementia onset before or within 1 year after parkinsonism onset [26]. Neuropathological lesions are similar in DLB and PD, with the involvement of synucleinopathy and Lewy bodies, but they do not share the same cerebral localization [30]. DLB neuropathology is frequently associated with AD pathology, particularly with the presence of plaques [1, 12].

The scientific literature on gender distribution in dementia traditionally reports a more pronounced prevalence of women in AD, and of men in PD and PDD [6, 22], with a 2:1 male/female ratio in PD. One study has shown a similar gender distribution between individuals with DLB (*n* = 487) and PDD (*n* = 297) [9], with male sex being more prevalent: 62.6% in DLB and 61.3% in PDD. Other studies in DLB showed a slight male predominance (in China, *n* = 58, 51.7% were male [31]; in a 5-year prospective cohort in Norway, *n* = 67, 47.8% were female [25]; in another Norwegian cohort, *n* = 72, 44.4% were female [5]). A U.S. study (*n* = 168,629) by Goodman et al. [11] revealed that the prevalence of DLB was higher among men, and Savica et al. [26] reported a higher incidence of DLB (*n* = 64) in men (4.8 vs 2.2 per 100,000 person-years). Blanc et al. reported a slight male predominance in DLB (*n* = 131, 52.2% were male) and a female predominance in AD (*n* = 1000, 34.9% were male) and in AD + DLB (*n* = 28, 43.6% were male) [4].

However, in a 2009 study of individuals with DLB in Italian memory clinics (*n* = 102), researchers did not report any gender differences in DLB [8]. In a retrospective cohort designed to study mortality, Price et al. showed a slight female predominance: 51.4% in the DLB group (*n* = 251) [23]. Authors of a review [29] evaluating the prevalence and incidence of DLB reported that eight studies included the gender of patients diagnosed with DLB. Of these, five reported disproportionately more females with the disease when controlling for the gender of the sample, and three reported disproportionately more males. The authors concluded that gender difference in DLB is still unclear, and future studies should report the gender of patients with DLB.

Regarding the relationships between gender distribution and neural correlates, the scientific literature reported a male/female ratio of 2.91 with a higher male predominance in neocortical “diffuse” DLB (*n* = 129) [19] than in “intermediate”/limbic subtype (*n* = 44), and a balanced sex ratio (male/female sex ratio, 1.14) in double diagnosis of DLB + AD (*n* = 470). Thus, DLB seems to be close to both AD and PDD clinically and neuropathologically, and the sex ratio in DLB is unclear, with a slight predominance of either men or women, whereas it appears that men are predominant in PDD and that AD is more common in women.

To better describe the epidemiologic characteristics of DLB, as well as to better characterize the putative links between the epidemiology and pathophysiology of a disease often considered an in-between pathology between AD and PD, it could be of interest to consider the gender distribution in DLB as an in-between epidemiologic characteristic of DLB. We aimed to investigate the gender distribution among individuals diagnosed with DLB followed in the French national cohort of people with AD and related disorders. Considering the generally low prevalence of DLB in the general population, studies published so far were conducted with limited sample sizes. According to the clinical insight that the features of DLB are mixed between AD and PD, and owing to conflicting previous results of sex ratios in DLB, we hypothesized that the gender distribution in DLB is balanced between male and female individuals as well as between AD and PDD or PD.

## Methods

### French National Alzheimer database

The French National Alzheimer database (Banque Nationale Alzheimer [BNA]) is part of the French strategy in its fight against dementia [20, 21, 27], and it has stored information from the end of 2009. The goal of this database is to provide information about the medical activities of the French memory centers in order to adapt the healthcare provision and generate epidemiologic knowledge about the diseases and the medical practices. Information collected in the BNA consists of a limited set of data concerning demographic, diagnosis, and clinical data selected by a national consensus group. The number of variables was restricted to facilitate and enhance care providers’ participation in this national database. Data are collected from 427 French memory units: the 399 memory centers (secondary level), the 28 resource and research memory centers (tertiary level), and 61 independent neurologists (primary care) who expressed willingness to participate.

Each time a patient consults a center, a record is generated and transferred to the database. Therefore, one patient can have several files in the BNA, depending on the number of medical visits he/she has had.

Variables used for this study were gender, age, living conditions, education level (with five levels according to the French education system and corresponding to the following categories: no schooling, primary school level [equivalent to 1–5 years of education], secondary school level with 6–9 years of education, secondary school level with 10–12 years of education, and university level [over 12 years of education]), type of center, referring modalities, location of the patient, Mini Mental State Examination (MMSE) score [10], date of consultation, diagnosis, and treatments. The BNA differentiates 38 diagnostic groups based on International Classification of Diseases, Tenth Revision, codes. The code related to DLB is G31.83, to AD is F00.1, to PDD is F02.3, and to PD is G20. More details on this database are described elsewhere [15].

### Study design and participant selection

A repeated cross-sectional study was conducted using data from the BNA from January 1, 2010, through December 31, 2015. Individuals who received one of the diagnoses of interest (AD, DLB, PDD, or PD) at least once during the study time frame were included in the analysis.

Individuals were included in the DLB group (or AD group) if the first DLB (or AD) diagnosis was made between 2010 and 2015 and if the last diagnosis was DLB (or AD). Individuals with a first PD diagnosis between 2010 and 2015 and with PD or PDD as their last diagnosis were included in the PD group. Individuals with a first PDD diagnosis between 2010 and 2015 with PDD as their last diagnosis and who never had a PD diagnosis were included in the PDD group. Checking the last diagnosis of the patient was intended to increase the reliability of the diagnosis.

Individuals who had the diagnosis of interest already when first registered in the database were included only if their first consultation for memory troubles was in the same year or the year before the first visit. This was intended to exclude patients who had a diagnosis established for a long time and to collect data at the time of the first diagnosis.

**Fig. 1:**
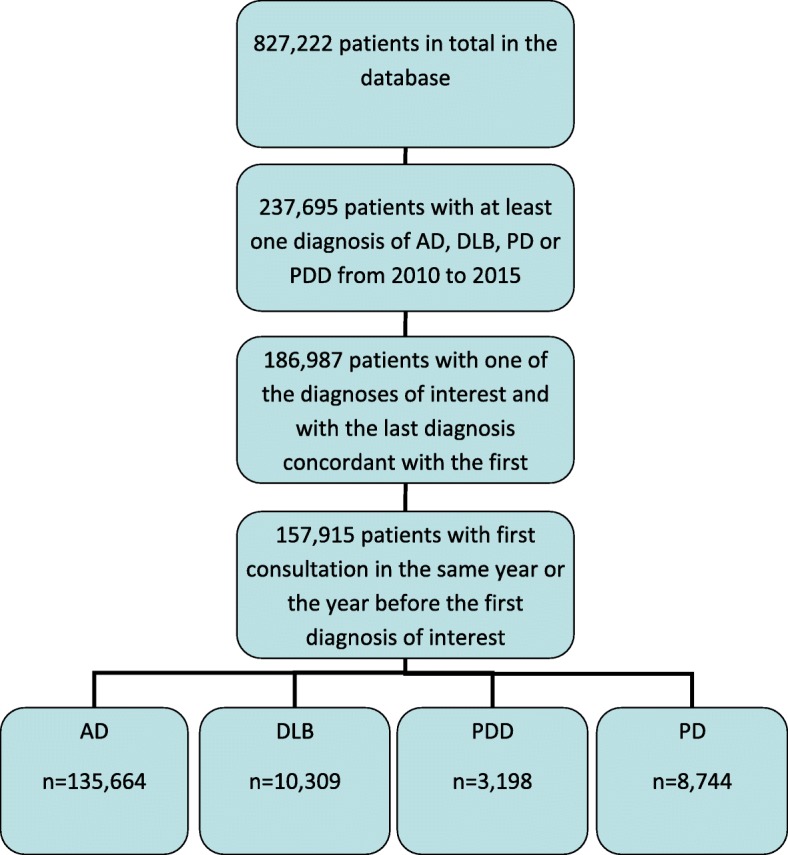
Selection of the participants included in the study

To describe the whole population included in the study, we selected data at the first diagnosis of interest. Given the importance of cognitive status, only patients with at least one existing MMSE evaluation at less than 1 year before or after the first diagnosis of interest were considered in the analysis (Fig. 1).

### Statistical analysis

Descriptive analyses were conducted using percent and frequency for qualitative variables and mean with SD for quantitative variables. Variables associated with diagnosis (i.e., AD, DLB, PD, and PDD) were tested using analysis of variance for quantitative variables and chi-squared tests for qualitative variables. The multivariate analysis testing relationship between diagnosis as the dependent variable and possible explicative variables such as sex ratio, age at the first diagnosis, MMSE, level of education, type of center, referring modality, living in the community, and living close to memory clinic was performed using multinomial logistic regression analysis. The DLB diagnosis was used as the reference modality.

A *p* value less than 0.05 was considered significant and kept in the final model. Adjusted ORs are presented with 95% CIs. All tests were performed bilaterally.

In addition to these usual multivariate analyses, and because of the large size of our cohort, we decided to run a second type of analysis: Bayesian analysis. This analysis was also performed as a simple way to deal with significantly labeled differences between large-sized groups. Bayesian analysis is an appropriate alternative to the frequentist methods. It is even thought to be more appropriate in many cases, such as with small samples or, on the contrary, large samples. Bayesian inference allows prior knowledge to be updated as data comes in for retrieving a posterior knowledge. The Bayes theorem used for inference multiplies prior distributions (what is known about a parameter before having data) by the likelihood (based on modeling assumptions). Specific techniques, Markov chains, and Monte Carlo integration are then used for having a posterior distribution for the parameter and some of its characteristics. Here we used a burn-in of 1000 iterations (to allow Markov chains to reach stationary distribution) and 4000 useful iterations for estimates. Note that a central 95% posterior interval (e.g., the interval between the 2.5% and 97.5% quantiles) of this sample can directly be interpreted as containing the true parameter with a high probability, unlike classical confidence intervals. We use this credible posterior interval as mimicking confidence intervals [14].

In our case, a major benefit of the hierarchical structure imposed by Bayesian inference is that the estimate of quantities (such as mean or percentage) between distinct groups undergoes “shrinkage” toward an overall mean. Furthermore, the Bayesian techniques allow acceptance of a null hypothesis (not only rejection), which is not only a comparison with 0 (for example, for a difference). In our case, a difference of 2 years for age, a difference of 2 points on the MMSE, and a difference of 0.05 in the proportion of women were considered significant. Furthermore, for example, considering the sample of posterior difference between mean ages in two groups, the proportion in these iterations greater than 2 is interpreted as the probability that this difference is greater than 2 in the population.

Statistical analyses were done with SAS Enterprise Guide software, version 5.1 (SAS Institute, Cary, NC, USA). Bayesian analyses were done with WinBugs 1.4 software.

## Results

### Population studied

According to our selection criteria, we assembled four groups: DLB (*n* = 10,309), AD (*n* = 135,664), PDD (*n* = 3198), and PD (*n* = 8744) (Fig. 1). Demographic characteristics of the study population are presented in Table 1. Individuals with a DLB diagnosis were aged 80.1 ± 7.8 years (mean ± SD) and individuals with AD, PDD, and PD were aged 81.4 ± 8.0, 79.5 ± 8.1, and 73.9 ± 10.8 years, respectively. For the three dementia groups (DLB, AD, and PDD), the majority of individuals were living in the community (more than 75%), within a 50-km distance from the memory clinic (more than 89%), were mainly seen by the general practitioner (GP) (more than 48%), and had a primary or lower education level (more than 47%).

**Table 1:** Descriptive characteristics of the four groups and comparisons (multinomial logistic regression)

	Univariate analysis								Multivariate analysis						
	AD (*n* = 135,664)		DLB (*n* = 10,309)		PDD (*n* = 3198)		PD (*n* = 8744)		AD		DLB	PDD		PD	
	Mean	[SD]	Mean	[SD]	Mean	[SD]	Mean	[SD]	Adjusted OR	[95% CI]	Adjusted OR	Adjusted OR	[95% CI]	Adjusted OR	[95% CI]
Age at first consultation with the diagnosis, years	81.42	[7.98]	80.11	[7.84]	79.45	[8.09]	73.86	[10.79]	1.01**	[1.01; 1.01]	1	0.99**	[0.99; 0.99]	0.97**	[0.96; 0.97]
MMSE at ± 1 year after diagnosis	17.71	[5.93]	18.48	[6.24]	19.18	[5.96]	24.26	[5.22]	0.98**	[0.98; 0.99]	1	1.03**	[1.02; 1.04]	1.26**	[1.25; 1.27]
	No.	(%)	No.	(%)	No.	(%)	No.	(%)	Adjusted OR	[95% CI]	Adjusted OR	Adjusted OR	[95% CI]	Adjusted OR	[95% CI]
Sex															
Female	95,098	(70.1)	5635	(54.7)	1452	(45.4)	3765	(43.1)	1.83**	[1.75; 1.92]	1	0.70**	[0.64; 0.76]	0.86**	[0.81; 0.93]
Male	40,566	(29.9)	4674	(45.3)	1746	(54.6)	4979	(56.9)	1		1	1		1	
Type of center															
Memory clinic	101,012	(74.5)	6712	(65.1)	2385	(74.6)	4798	(54.9)	1		1	1		1	
Regional specialized memory clinic	31,320	(23.1)	3226	(31.3)	695	(21.7)	3508	(40.1)	0.76**	[0.73; 0.80]	1	0.54**	[0.48; 0.60]	0.87**	[0.80; 0.94]
Private practice neurologist	3332	(2.5)	368	(3.6)	118	(3.7)	438	(5.0)	0.66**	[0.59; 0.75]	1	0.95	[0.75; 1.20]	1.18*	[1.00; 1.40]
Education															
No education	11,075	(8.2)	663	(6.4)	194	(6.1)	334	(3.8)	1.22**	[1.09; 1.36]	1	1.20	[0.95; 1.52]	1.88**	[1.57; 2.26]
Primary	64,430	(47.5)	4484	(43.5)	1327	(41.5)	2471	(28.3)	1.06	[0.98; 1.14]	1	1.16	[0.99; 1.35]	1.25**	[1.11; 1.39]
Secondary first cycle	21,516	(15.9)	1759	(17.1)	517	(16.2)	1595	(18.3)	1.04	[0.96; 1.14]	1	1.07	[0.90; 1.28]	1.19**	[1.05; 1.34]
Secondary second cycle	11,971	(8.8)	945	(9.2)	241	(7.5)	1027	(11.8)	1.11*	[1.01; 1.23]	1	1.00	[0.82; 1.23]	1.28**	[1.11; 1.47]
Superior	10,510	(7.8)	1110	(10.8)	317	(9.9)	1308	(15.0)	1		1	1		1	
Unknown	16,162	(11.9)	1348	(13.1)	602	(18.8)	2009	(23.0)	1.00	[0.91; 1.11]	1	1.92**	[1.59; 2.30]	2.78**	[2.44; 3.18]
Initially referred by															
General practitioner	90,354	(66.6)	5740	(55.7)	1562	(48.8)	3917	(44.8)	1		1	1		1	
Neurologist	7876	(5.8)	1118	(10.8)	526	(16.5)	2319	(26.5)	0.56**	[0.52; 0.61]	1	1.72**	[1.49; 1.99]	1.98**	[1.79; 2.19]
Other specialists	14,136	(10.4)	1406	(13.6)	392	(12.3)	679	(7.8)	0.67**	[0.63; 0.72]	1	1.05	[0.91; 1.21]	0.75**	[0.66; 0.84]
Direct	5807	(4.3)	389	(3.8)	105	(3.3)	343	(3.9)	0.96	[0.85; 1.08]	1	1.11	[0.87; 1.42]	1.02	[1.85; 1.23]
Others	17,488	(12.9)	1656	(16.1)	613	(19.2)	1486	(17.0)	0.65**	[0.61; 0.70]	1	1.32**	[1.16; 1.49]	1.82**	[1.65; 2.01]
Community-dwelling															
No	22,756	(16.8)	2045	(19.8)	778	(24.3)	1239	(14.2)	1		1	1		1	
Yes	112,908	(83.2)	8267	(80.2)	2420	(75.7)	7505	(85.8)	1.37**	[1.29; 1.45]	1	0.71**	[0.63; 0.79]	0.79**	[0.71; 0.87]
Location of the patient															
Within 50 km from the memory clinic	124,519	(91.8)	9220	(89.4)	2899	(90.7)	6816	(78.0)	1.08*	[1.00; 1.16]	1	0.97	[0.84; 1.13]	0.61**	[0.55; 0.67]
> 50 km from the memory clinic	11,145	(8.2)	1089	(10.6)	299	(9.4)	1928	(22.0)	1		1	1		1	

*Abbreviations: AD* Alzheimer’s disease, *DLB* Dementia with Lewy bodies, *PDD* Parkinson’s disease dementia, *PD* Parkinson’s disease, *MMSE* Mini Mental State Examination

** *p* < 0.01; *p < 0.05

### Sex ratio

**Fig. 2:**
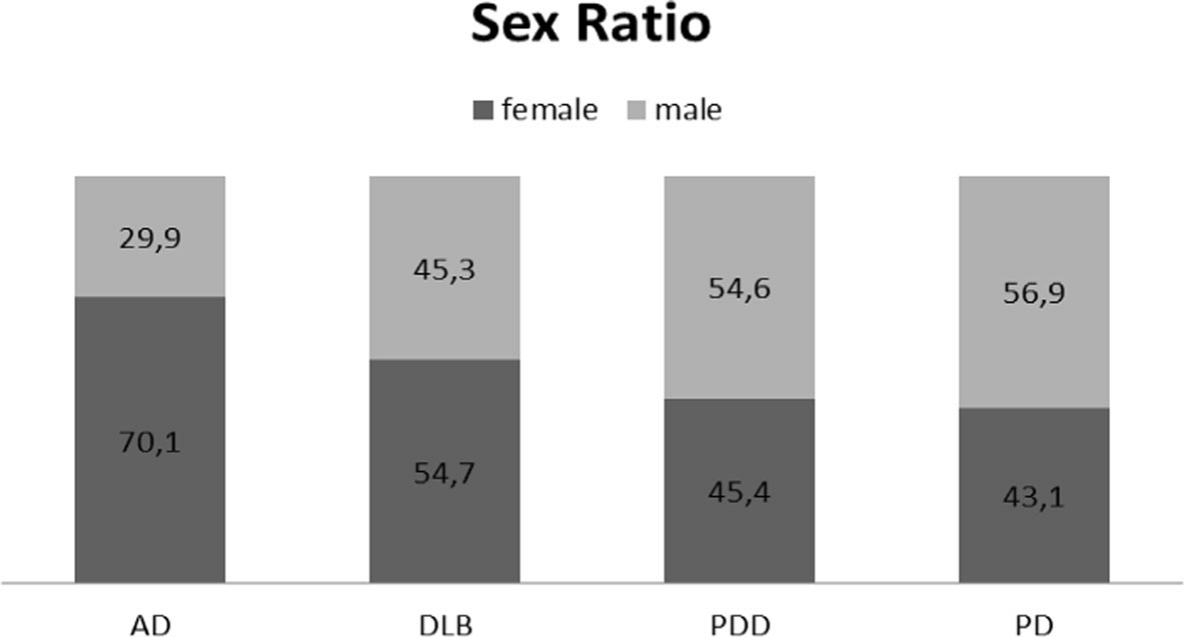
Sex ratio. Percentage of female and male participants of the four groups

The proportion of women according to each diagnostic group was distributed as follows (using univariate analysis): 54.7% (sex ratio = 1.21) for DLB, 70.1% (sex ratio = 2.34) for AD, 45.4% (sex ratio = 0.83) for PDD, and 43.1% (sex ratio = 0.76) for PD (Fig. 2). Multinomial logistic regression multivariate analysis showed significant differences in sex ratio between each group compared with DLB (Table 1). Given the large number of subjects in each subgroup, we also used a Bayesian analytical method to investigate clinically relevant differences between each group, in addition to the multivariate analysis (Table 2). Our results showed that gender distribution was significantly different (difference of 5% defined before analyses) between each group, except between PD and PDD. We found a female predominance in AD and a male predominance in PD and PDD; among DLB subjects, there was a slight predominance of women. An age-stratified analysis of sex ratio in DLB was also conducted (Table 3). We observed that for people younger than 75 years old, more males than females had DLB. For people older than 75 years of age, DLB was more common in females, and the sex ratio in favor of females increased with age.

**Table 2:** Comparison of descriptive characteristics of the four groups (Bayesian analyses)

	DLB/AD	DLB/PDD	DLB/PD	AD/PDD	AD/PD	PDD/PD
Probability to get a difference of 5%						
Sex	1	1	1	1	1	0.004
Community-living	0	0.278	0.886	1	0	1
Location of the patient	0	0	1	0	1	1
Center: tertiary level vs secondary and primary levels	1	1	1	0	1	1
Probability to get a difference of						
2 years for the age	< 0.001	< 0.001	1	0.436	1	1
2 points for MMSE	< 0.001	< 0.001	1	< 0.001	1	1

*Abbreviations: AD* Alzheimer’s disease, *DLB* Dementia with Lewy bodies, *PDD* Parkinson’s disease dementia, *PD* Parkinson’s disease, *MMSE* Mini Mental State Examination

The probability corresponds to the numbers of iterations where the difference of 5% for qualitative variables and 2 points for quantitative variables were observed among all completed iterations

A probability = 1 means all iterations show the difference whereas a probability = 0 means no iteration shows the difference

**Table 3:** Sex ratio in dementia with Lewy bodies according to age

	DLB	
	No.	(%)
Age at first consultation with the diagnosis < 75 years		
Female	828	(38.6)
Male	1315	(61.4)
Age at first consultation with the diagnosis 75–80 years		
Female	1287	(51.9)
Male	1195	(48.1)
Age at first consultation with the diagnosis 80–85 years		
Female	1680	(56.8)
Male	1280	(43.2)
Age at first consultation with the diagnosis ≥85 years		
Female	1840	(67.6)
Male	884	(32.4)

*DLB* Dementia with Lewy bodies

### Other clinical features

Other variables, such as MMSE, age, type of center, education level, referring modalities, living conditions, and location of the patient were included in the multinomial logistic regression (Table 1). Multivariate analyses showed significant differences for each group compared with DLB on age at first diagnosis, MMSE, and living conditions. Regarding the type of center in which the patient consults, there was also a significant difference between each group (except for PDD and DLB when referred to a private practice neurologist).

Regarding age at the first diagnosis, with the Bayesian method (2 years of difference was estimated to be clinically relevant), there was a difference between DLB and PD but not between DLB and AD or PDD (Table 2). Patients with PD are significantly younger than those in the three other groups (DLB, AD, and PDD).

For MMSE in the year of the first diagnosis (a difference of 2 points was decided), subjects diagnosed with DLB did not show any difference compared with subjects diagnosed with AD or PDD. In PD, MMSE was significantly different from the three other groups. The MMSE (mean ± SD) in the PD group was higher (24.3 ± 5.2) than in the DLB (18.5 ± 6.2), AD (17.7 ± 5.9), and PDD (19.2 ± 6.0) groups.

DLB had an intermediary proportion of living in the community (80.2%) between AD (83.2%) and PDD (75.7%). Patients with PD were more likely to be living in the community than those in the other groups (85.8%), but there was no significant difference between DLB and AD or PDD (Table 2). A significant difference was found for DLB compared with PD only and for patients with PDD compared with those with AD and those with PD.

The comparison of the type of center (tertiary level vs secondary level-primary care) showed a significant difference between each group, except between AD and PDD, where no difference was shown. Compared with patients with AD and PDD, patients with DLB were more often cared for in a specialized center (tertiary level). The repartition between the five education levels was similar between the three dementia groups. Patients with PD more often had a higher education level.

## Discussion

### Gender distribution among diagnostic groups

The main finding of the present study was the slight predominance of women in the DLB population, whereas there was a strong predominance of women in the AD population, and men were slightly more frequent among PDD individuals. These significant differences are confirmed using a Bayesian analysis method, whereas PD and PDD did not show any difference in gender distribution.

The gender distribution (female/male ratio) for PD and AD is consistent with previous studies (around 0.67 and 2, respectively) [7, 24]. The gender distribution in PDD is less studied and has been reported to be around 0.5 (the female predominance was higher in the BNA cohort at 0.83) [22], but sex (male) as a predictive factor of developing dementia in PD is still discussed [22].

The strength of the present study is the size of the cohort, contrary to the majority of previous studies based on more limited populations. Several clinical or anatomopathological studies have shown a male predominance in DLB; however, others found a slight predominance of women, as in our study. Our result might be due to the high mean age of our population (80.1 years). To explain their results, Zahirovic et al. [32] relied on previous studies that had shown higher prevalence of DLB in men in the age group 70–79 years and more balanced prevalence in age groups older than 80 years. Indeed, we observed in our study that, contrary to younger people, for those older than 75 years, DLB is more common in females, and the sex ratio in favor of females increases with age.

In our study, diagnoses were made by clinical judgment and not according to anatomopathological results. Nelson et al. [19] showed that clinically suspected DLB tended to be underdiagnosed in men and overdiagnosed in women. This might explain the predominance of women in our study, which reflects clinical practice.

The recent concept of DLB associated with AD should be taken into account. The sex ratio might be balanced between AD and PDD because of an association of DLB + AD in the group of DLB in our clinical cohort. In an autopsy study [18], females were more predominant in the DLB + AD population than in pure DLB: 48% vs 32.2% in one cohort and 63.2% vs 28.6% with female predominance in the DLB + AD group in the other cohort. Concordant with these results, a clinical study comparing AD, DLB, and AD + DLB patients [4] showed that females are predominant in AD and in AD + DLB, whereas males are predominant in DLB. Furthermore, in this study, AD + DLB patients were older than patients in the two other groups. So, older DLB patients seem to more often be female and could have a more mixed pathology of AD + DLB. Anatomopathological studies would be very informative but are not easy to conduct and would thus include fewer subjects than in our study.

DLB is a disease in between AD and PDD, clinically and biologically, for the following reasons:

- Clinically because DLB shares clinical symptoms with AD (cognitive impairment with executive and memory dysfunction, outcomes of the cognitive dysfunctions [4]) and also with PDD (rigidity, akinesia, cognitive impairment with executive and visual constructive dysfunction, RBD, and neuroleptic sensitivity [13])
- Biologically because the cerebrospinal fluid AD profile is more common in DLB than in PDD, gray matter atrophy is more frequent and more severe in DLB and AD than in PDD [2, 3], amyloid-β load and Tau load are more severe and more extended in DLB than in PDD [13], but inversely alpha-synuclein load is more important in DLB and PDD

Thus, it seems logical that in DLB, the sex ratio, probably specific to each disease, is not the same as in AD or in PD or PDD, but in between, with a balanced sex ratio. This finding strengthens the arguments that DLB is a distinct disease from PDD and AD.

### Clinical characteristics at first diagnosis

Analysis showed differences between DLB, AD, and PDD on age and MMSE at first diagnosis. The observed differences were very mild, however, and likely not relevant. Indeed, our Bayesian analysis only showed differences with the PD subgroup in which patients were younger and had a higher MMSE.

Community-living individuals in the DLB population were less frequent than in AD but more frequent than in PDD. The differences are not very important, however, and were not confirmed in Bayesian analysis. Patients with DLB were more often referred to research memory centers (regional level, tertiary center) than patients with AD or PDD.

### Limitations of the present study

The BNA represents a valuable epidemiologic tool because it grants access to many patients with dementia and permits follow-up studies. Although the size and the follow-up information for the dementia population make the BNA a unique database, several limitations should be noted.

First, data are entered into the BNA by different physicians; despite the fact that they all follow standard criteria for diagnosis, there is no external validation that those criteria were met, and this may decrease the reliability of the diagnosis code assigned to patients. Diagnosis of DLB using the current criteria is very specific (specificity around 90–100%) [16]. It could thus be assumed that a DLB diagnosis made in the BNA by a physician is reliable, especially because data are registered by specialized centers. Additionally, we did not take into account patients for whom diagnosis of DLB changed during follow-up, indicating possible atypical diagnosis of DLB. Thus, we can consider that, according to clinical practice, patients in this study represent the DLB population.

Second, individuals included in the BNA are not fully representative of the total French population with AD and associated disorders; indeed, the BNA includes the great majority of individuals with AD and associated disorders who are referred to specialized centers (French memory units), but one part of the population with dementia is under GP supervision only (GPs do not currently have access to the BNA), and another part of the population is referred to specialists (geriatricians, neurologists, psychiatrists) who are not using the BNA database. This could be particularly true for individuals with DLB, among whom the probability of consulting in classical neurologic settings for parkinsonism or in psychiatric settings for psychotic symptoms may increase.

## Conclusions

Our study shows that the sex ratio is balanced in DLB, with a slight predominance of females, and intermediate between the sex ratios in AD and PDD. This strengthens arguments that DLB is a disease distinct from PDD and AD, even if symptoms can be close, and that DLB and AD may be associated. Autopsy studies would be helpful to confirm this differences in sex ratios between the three diseases.

## Abbreviations

AD: Alzheimer’s disease
BNA: Banque Nationale Alzheimer
DLB: Dementia with Lewy bodies
GP: General practitioner
MMSE: Mini Mental State Examination
PD: Parkinson’s disease
PDD: Parkinson’s disease dementia
RBD: REM sleep behavior disorder
